# Three endogenous pararetrovirus genomes in *Medicago sativa*

**DOI:** 10.1128/mra.00742-24

**Published:** 2024-09-27

**Authors:** Raad Mohammed Ali, Osamah Alisawi, Maadh Al Fahad

**Affiliations:** 1 Plant Protection Department, Faculty of Agriculture, University of Tikrit, Tikrit, Iraq; 2 Plant Protection Department, Faculty of Agriculture, University of Kufa, Najaf, Iraq; Department of Biology, Queens College, Queens, New York, USA

**Keywords:** alfalfa genome, bioinformatics, endogenous pararetroviruses, whole-genome sequencing

## Abstract

The genome sequences of three related endogenous pararetroviruses were obtained by high-throughput genomic sequencing of *Medicago sativa*. The genomes were found to be integrated within plant genes. The phylogeny revealed that Caulimovirus-MSa3 was closely related to caulimoviruses of petunia, whereas Caulimovirus-MSa1 and Caulimovirus-MSa2 were distinct from constructed clades.

## ANNOUNCEMENT

**Fig 1:**
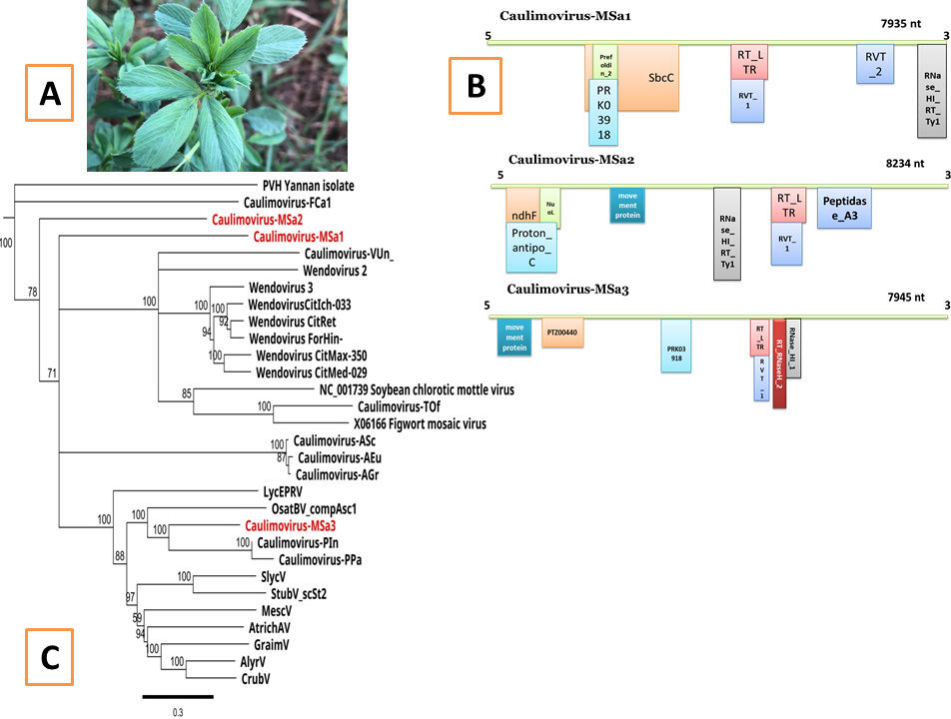
Leaves of the alfalfa plant that was sampled for DNA extraction (**A**). Genome organization of each sequence of the detected viral elements showing a particular pattern for each integrant (**B**). The phylogeny indicates that Caulimovirus-MSa3 was closely related to Caulimovirus-Pin and Caulimovirus-PPa of petunia, while Caulimovirus-MSa1 and Caulimovirus-MSa2 were positioned out of constructed clades. The neighbor-joining tree was built by the Geneious tree builder version 2024.0.5, and alignments of full-genome nucleotide sequences were performed with ClustalW. Based on the Hasegawa-Kishino-Yano substitution model, phylogenies of alignments were inferred with 1,000 bootstraps to assess node support. The sequences of Wendovirus CitMax-350, Wendovirus CitMed-029, Wendovirus CitRet, Wendovirus ForHin-, WendovirusCitIch-033, Wendovirus 2, and Wendovirus 3 were taken from reference (13). The sequences of SlycV, AlyrV, AtrichAV, GraimV, CrubV, OsatBV_compAsc1, StubV_scSt2, and MescV were obtained from Geering et al (11). The sequences of Caulimovirus-TOf, Caulimovirus-FCa1, Caulimovirus-VUn, Caulimovirus-ASc, Caulimovirus-AGr, Caulimovirus-AEu, Caulimovirus-PPa, and Caulimovirus-Pin were downloaded from Repbase data set. The sequences of soybean chlorotic mottle virus (NC_001739) and figwort mosaic virus (X06166) were taken from GenBank. The sequence of LycEPRV was downloaded from GenBank under accession number DQ273262. The outgroup member was *Potato virus* H (**C**).

Plant genomes contain endogenous caulimovirids, a class of transposable elements positioned in non-coding regions of such genomes. They were created by the integration of viral DNA into chromosomes millions of years ago during ancient infections (1). Endogenous pararetroviruses in plants were recently detected through genomic sequencing (2). They are found in partial or entire sequences integrated into particular positions within chromosomes and inherited over generations (3). The alfalfa genome was not previously reported to contain these elements, except soybean chlorotic mottle virus and figwort mosaic virus-like endogenous sequences that were identified segmented within tetraploid and diploid genomes of alfalfa by Boutanaev and Nemchinov (4). From a local variety of alfalfa leaves collected in Balad region, Saladin Province, Iraq on 4 October 2023, DNA was extracted using cetyl-trimethylammonium bromide (5) in DNA Link company in the Republic of Korea (Fig. 1A). To prepare NGS libraries for DNA sequencing, TruSeq DNA Library Preparation Kit was used. DNA sample was examined using the Novaseq6000 2 × 150 bp reads technique with the WGS application (PCR Free550). The reads were trimmed by Trimmomatic-0.39 (6) and BBDuk version 37.22 in Geneious Prime 2024.0.5 (7) programs. The data were explored in the RepeatExplorer pipeline (8). To identify taxonomic and protein domains, RepeatExplorer2 (Galaxy 2.3.8.1) was applied, and viridplantae version 3.0 was selected. The cluster was imported into the Repbase dataset 2024 (https://www.girinst.org/) (9) and the Basic Local Alignment Search Tool for further identification (10). The data were mapped against each cluster more than six runs to obtain the estimated full sequence (between 7.2 and 8.5 kbp) (11) using the Geneious DNA mapper (Sensitivity: Medium-Low Sensitivity) and then aligned with previously published endogenous pararetroviruses. For annotation, BLASTx version 5 (12) and Open Reading Frame Finder in Geneious Prime 2024.0.5 were also used. Default parameters were used unless otherwise specified. As a result, 106,701,106 clean and paired-end reads were generated, and three viral elements belonging to the *Caulimoviridae* family and *Caulimovirus* genus were identified in the alfalfa genome named Caulimovirus-MSa1, Caulimovirus-MSa2, and Caulimovirus-MSa3. Caulimovirus-MSa1 was assembled with 68,281 reads, 1,299 copies, and 1,070 coverage depth, and 60,469 reads were assembled for Caulimovirus-MSa3 with 1,149 copies and 1,949 coverage depth. In contrast, 252,257 reads were assembled for Caulimovirus-MSa2 with 4,626 copies and 18,064 coverage depth. The lengths were 7,935, 8,234, and 7,945 bp with GC contents of 39.7%, 29.2%, and 30.6%, respectively. Domains from plant genes and retrotransposons were found to be involved in the integrant genomes. The Caulimovirus-MSa1 encodes seven coding regions: Prefoldin, PRK03918, SbcC, RT_LTR, RVT-1, RVT-2, and RNase HI. The Caulimovirus-MSa2 has eight domains of ndhF, Protonantipo, NuoL, Movement protein, RNase_HI, RT_LTR, RVT-1, and Peptidase-A3. Caulimovirus-MSa3 has seven domains of Movement protein, PTZ00440 (reticulocyte binding protein 2-like protein), PRK03918, RT_LTR, RVT_1, RT_RNaseH_2, and RNase_H1 (Fig. 1B). The phylogeny shows that Caulimovirus-MSa3 was closely related to Caulimovirus-Pin and Caulimovirus-PPa of petunia, whereas Caulimovirus-MSa1 and Caulimovirus-MSa2 were distinct from constructed clades. The result confirmed the existence of such elements that belong to the *Caulimovirus* genus and related genomes (Fig. 1C).

## Data Availability

This Whole Genome Shotgun project of alfalfa has been deposited in GenBank under accession no. SRR28886118. The sequences of Caulimovirus-MSa1, Caulimovirus-MSa2, and Caulimovirus-MSa3 have been deposited in GenBank under accession numbers PP874930, PP874931, and PP889324, respectively.
